# Long-Term Impact of N, P, K Fertilizers in Different Rates on Yield and Quality of *Anisodus tanguticus* (Maxinowicz) Pascher

**DOI:** 10.3390/plants12112102

**Published:** 2023-05-25

**Authors:** Kaiyang Chen, Lei Ma, Chen Chen, Na Liu, Bo Wang, Yuying Bao, Zhengrong Liu, Guoying Zhou

**Affiliations:** 1 Key Laboratory of Tibetan Medicine Research, Northwest Institute of Plateau Biology, Chinese Academy of Sciences, Xining 810008, China; 2 University of Chinese Academy of Sciences, Beijing 100049, China; 3 Qinghai Research and of Environmental Sciences, Xining 810008, China; 4 College of Agriculture and Animal Husbandry, Qinghai University, Xining 810016, China

**Keywords:** “3414” fertilizer, equation, rational cultivation, yield, quality

## Abstract

*Anisodus tanguticus* (Maxinowicz) Pascher (Solanaceae) is a traditional Chinese herb that is widely used in folklore and clinical practice. In recent years, wild populations have been severely impacted to the point of extinction due to over-harvesting and reclamation. Therefore, artificial cultivation is important to relieve the pressure of market demand and protect wild plant resources. Using a “3414” fertilization design, i.e., 3 factors (N, P, and K), 4 levels, and 14 fertilization treatments, with 3 replicates and a total of 42 experimental plots, *A. tanguticus* was harvested in October 2020, June 2021, August 2021, and October 2021, and the yield and alkaloid content were determined. The study aimed to provide a theoretical basis and technical reference for the standardization of *A. tanguticus* cultivation. Biomass accumulation and alkaloid content showed a trend of increasing and then decreasing with the application of nitrogen, phosphorus, and potassium, and the biomass accumulation was the highest at the application levels of nitrogen and phosphorus in T6 and T9 and at the application levels of medium and low potassium. The alkaloid content showed an increasing trend between October of the first year and June of the second year and a decreasing trend in the second year with the increase in the harvesting period. Yield and alkaloid yield showed a decreasing trend between October of the first year and June of the second year and an increasing trend in the second year with the increase in the harvesting period. The recommended application rates are 225–300 kg/ha^2^ for nitrogen, 850–960 kg/ha^2^ for phosphorus, and 65–85 kg/ha^2^ for potassium.

## 1. Introduction

*Anisodus tanguticus* (Maxinowicz) Pascher is distributed across eastern Tibet [1], Qinghai, Sichuan, Gansu, and other places in China [2,3]. *A. tanguticus* root is used for medicinal purposes [4]. Its active ingredients are mainly tropane alkaloids, such as scopolamine, anisodamine [5], atropine, and anisodine [6], which have anesthetic and antispasmodic properties and analgesic [3], sedative, anti-phosphorus poisoning [7], and other effects [8]. The aboveground part is mixed into cattle feed, which has the effect of fattening [9]. *A. tanguticus* is the most commonly used Tibetan medicine in China [8]. Its active ingredient, anisodamine [10], is listed as the first natural chemical drug developed by China in the world [11]. Its pharmacological and clinical effects are also recognized and valued by the world medical community [12]; for example, anisodamine and atropine can reduce glandular secretion and pulmonary edema [13]. Scopolamine has a sedative effect on the central nervous system and is clinically used as an anti-corona drug [14]. Anisodine hydrobromide combined with gastrodin can treat cognitive dysfunction in the elderly [15]. With the increase in clinical demand and functional development of tropane alkaloids, its market demand has steadily expanded year by year, and the artificial planting area has also continued to expand [16]. As with other medicinal plant production [17], fertilization has become an important measure to improve the growth characteristics and yield of *A. tanguticus* [6].

Nitrogen (N), phosphorus (P), and potassium (K) are essential nutrients for plant growth and development and are components of important organic compounds [18]. They also play an important role in various growth and development and physiological metabolism processes [19]. N, P, and K fertilization can improve seedling quality [20] and stress resistance by promoting plant growth and biomass accumulation [21], but the types and concentrations of N, P, and K limit the growth state [22] and rate of plants [23,24]. In recent years, the “3414” design has been gradually used in the production of medicinal plants [25]. For example, the compound application of 3414 effectively improved the quality of the Bupleurum root [26]. In addition, the “3414” fertilizer design has also been tested on a variety of Chinese herbal medicines such as *Panax notoginseng*, *rhubarb*, and *atractylodes* [27], and the effect is remarkable [28]. Therefore, rational fertilization can provide a theoretical basis for standardized cultivation and achieve the goal of high-yield and high-quality plants. Currently, the research on the cultivation of *A. tanguticus* mainly focuses on the relationship between the elements and alkaloids in the root with the altitude gradient [29], and the comparison of the active ingredient contents of wild and cultivated *A. tanguticus* [30]. The research on the regularity of fertilizer requirements and formula fertilization has not been reported yet.

The “3414” fertilizer effect test program is the recommended program design in the “Revised Draft of Technical Specifications for Soil Testing and Formula Fertilization (Trial) ”of the Ministry of Agriculture [31]. The three factors of N, P, and K are used in 3414, and there are four levels: 0, 1, 2, and 3. Among them, the level of 0 represents no fertilization, the level of 2 generally refers to the best local fertilization amount, the level of 1 is 0.5 times the level of 2, and the level of 3 is 1.5 times the level of 2. To meet the professional requirements of decision making, the fertilizer effect function method can be used to reasonably determine the application amount of N, P, and K based on soil fertilizer supply capacity, crop fertilizer demand law, and fertilizer effect [32]. Compared with other fertilization methods [33], the “3414” fertilization concept adopts an incomplete orthogonal regression design as a three-factor and four-level fertilization experiment [34]. It has the advantages of complete factors, multiple levels, simple operation, and convenient analysis, which meet the professional requirements of fertilization decision making [35].

This study aims to solve the problem of non-standard cultivation of *A. tanguticus* and provide a theoretical and practical basis for high-quality cultivation of *A. tanguticus*. The scientific basis of this study provides data support for the theory and practice of *A. tanguticus* fertilization and promotes the sustainable development of the *A. tanguticus* industry.

## 2. Results

### 2.1. Effects of Different Fertilization Methods and Harvest Periods on Dry Biomass of A. tanguticus

Different fertilizer application ratios significantly affected the underground yield of *A. tanguticus* (Table 1), which reached a maximum underground yield under the T6 (N2P2K2) treatment at the maturity stage in October of the first year and at the growth and maturity stages in August and October of the second year, and a maximum underground yield under the T9 (N2P2K1) treatment at the re-greening stage in June of the second year, with higher underground yields than T1 (control) for all fertilizer treatments. Under all N, P, and K fertilization conditions, underground yield showed a decreasing trend from October of the first year to June of the second year, and an increasing trend in the second year with an increasing harvesting period, reaching a maximum at maturity in October of the second year. Aboveground production of *A. tanguticus* decreased from October 2020 to June 2021, rose from June to August 2021, and decreased from August to October. Aboveground yield was highest during the second year of growth (August 2021).

**Figure 1:**
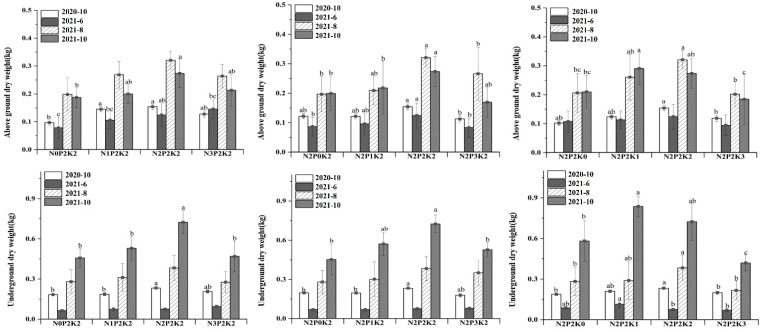
Effects of NPK single-factor fertilization on yield (kg/plant). Different letters in columns indicate significant differences according to Duncan’s test at α = 0.05).

In the single-variable treatment, the aboveground and underground yields under different nitrogen, phosphorus, and potassium fertilization levels showed a trend of increasing first and then decreasing with the increase in fertilization amount. As an exception, T11 reached the maximum value in June 2021, and it reached the maximum value at the medium fertilization level. Low potassium treatment showed a yield advantage in the whole growth and development process of *A. tanguticus*. In the second year of *A. tanguticus* growth, there was no significant difference between the T9 treatment and the T6 treatment (Figure 1).

### 2.2. Effects of Different Fertilization Methods and Harvest Periods on Alkaloid Content of A. tanguticus

The effects of different levels of NPK fertilizers on the contents of anisodine, anisodamine, scopolamine, and atropine in *A. tanguticus* were analyzed based on N2P2K2. The effects of NPK treatments on the contents of each alkaloid at the same harvesting period were significant, and the differences in the contents of alkaloids at different harvesting periods under the same treatment were also significant, and the contents in general showed a trend of increasing and then decreasing with the increase in fertilizer application. The contents of the four alkaloids showed an increasing trend from the maturity period in October 2020 to the re-greening period in June 2021, and in 2021, the contents of anisodine, anisodamine, and atropine showed a decreasing trend as the harvesting period of *A. tanguticus* increased, all with the highest contents in June. Scopolamine content increased from June to August and decreased from August to October in 2021, with the highest content in August (Table 2).

**Figure 2:**
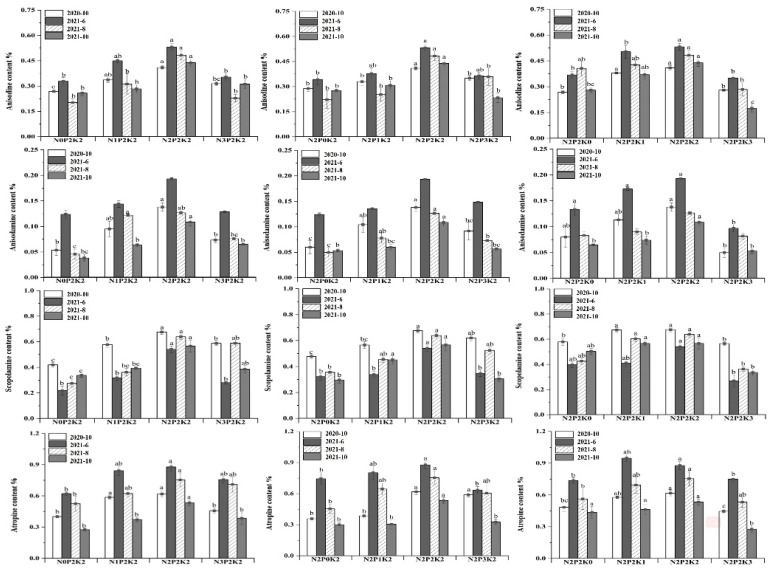
Effects of NPK single-factor fertilization on the alkaloid content. Different letters in columns indicate significant differences according to Duncan’s test at α = 0.05).

In terms of single-factor effect treatment, different fertilization treatments showed different degrees of promoting effect on the alkaloid content of *A. tanguticus* (Figure 2). Low potassium treatment (N2P2K1) showed a promotion of alkaloid content throughout the growth period of *A. tanguticus*, while high potassium treatment (N2P2K3) showed significant inhibition. The best fertilizer ratio with the highest alkaloid content in different harvest periods within two years was N2P2K2.

### 2.3. Effects of Different Fertilization Methods and Harvest Periods on Total Alkaloid Yield

**Figure 3:**
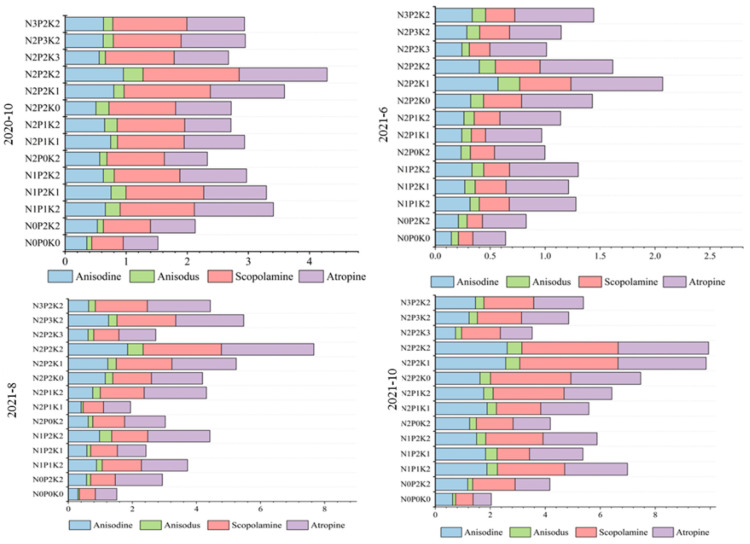
The total yield of alkaloids in different harvesting periods (kg/plant).

The accumulation of alkaloids under different fertilization conditions was observed, and the total alkaloids decreased from October of the first year to June of the second year. In the second year, the alkaloid content accumulated gradually with the increase in the harvesting period, and the highest value was obtained at the maturity stage of the second year (October 2021). The average alkaloid yields of the 14 fertilization treatments were 2.8 g/plant at the maturity stage in the first year, 1.02 g/plant at the re-greening stage in the second year, 3.21 g/plant at the growth stage, and 4.76 g/plant at the maturity stage. From the accumulation of alkaloids under different fertilization conditions, the alkaloid yield was lowest for N0P0K0 and highest for N2P2K2, which increased 182%, 152.67%, 406.8%, and 363.8%, respectively, during the four harvesting periods compared with no fertilization (Figure 3).

### 2.4. Range Analysis of Important Indexes under N-P-K Fertilization

The effect of different fertilization methods on the main indicators of *A. tanguticus* was analyzed by using the analysis of extreme differences. The effects of N, P, and K fertilization on biological indicators were different at different harvesting stages, as determined by categorical analysis of N, P, and K fertilization. Potassium fertilization had the greatest effect on anisodine, nitrogen fertilization had the greatest effect on anisodamine, and phosphorus fertilization had the greatest effect on scopolamine and atropine in the four harvesting periods. Nitrogen fertilization had the greatest effect on yield (underground dry weight and aboveground dry weight) during the regrowth period in June and the growing period in August 2021, and potassium fertilization had the greatest effect on yield (underground dry weight and aboveground dry weight) during the maturity period in October for annual and biennial *A. tanguticus*.

### 2.5. Interaction Analysis of N-P-K Fertilizer Applications

The yield of total alkaloids was the highest in October 2021. The fertilizer efficiency equation was fitted to obtain the best fertilization ratio and the highest yield/alkaloid content in October 2021. We used N × P, N × K, and P × K to obtain the binary quadratic equation and N × P × K to fit the ternary quadratic equation, and we took the first-order derivative of this equation to determine the highest biological alkali content. The specific fertilization ratio is shown in Table 3. Anisodine can reach the highest content of 0.401% under the conditions of N fertilizer 222.806 kg/ha^2^, P fertilizer 683.18 kg/ha^2^, and K fertilizer 84.9 kg/ha^2^. Anisodamine can reach the highest content of 0.081% under the conditions of N fertilizer 225.421 kg/ha^2^, P fertilizer 890.069 kg/ha^2^, and K fertilizer 75.507 kg/ha^2^. Scopolamine can reach the highest content of 0.534% under the conditions of N fertilizer 295.638 kg/ha^2^, P fertilizer 854.06 kg/ha^2^, and K fertilizer 67.135 kg/ha^2^. The highest content of atropine is 0.504% under the conditions of N fertilizer 300.214 kg/ha^2^, P fertilizer 930.203 kg/ha^2^, and K fertilizer 66.782 kg/ha^2^. The highest contents of the four alkaloids all tended to be found at the ratio of medium N, medium P, and low K fertilization (Table 4).

## 3. Discussion

There are many factors affecting the yield and quality of medicinal materials [36]. These factors include not only the genetic characteristics of medicinal materials [37], but also external factors such as the harvest period, geographical environment, soil, and climate of medicinal materials [38]. The growth habits of medicinal plants vary widely [39]. It is necessary to comprehensively consider the accumulation rules of active ingredients in medicinal materials, different medicinal parts, and environmental conditions [40,41]. In the process of harvesting [42,43], it is also necessary to take into account the yield and the content of active ingredients [44].

### 3.1. Effects of Different Fertilization Methods and Harvest Periods on Yield

In the present study, there were significant differences in the effect of each formulation fertilization treatment on yield at different developmental periods, but all treatments showed different degrees of increase compared to the blank group N0P0K0. *A. tanguticus* has a greater demand for N fertilizer at the greening, growing, and maturity stages, and the promotion effect of N is more pronounced in the early growth stage, while the promotion effect of phosphorus is more effective in the middle and late stages, and the dependence on K is not high throughout the reproductive period. Throughout the reproductive period, potassium application treatment K2 was the best promoter of *A. tanguticus* root yield, followed by K1, which was related to the need to accumulate large amounts of starch during the tuber growth period [45,46]. Excessive potassium fertilization was not conducive to tuber expansion [47] and starch accumulation [48]. This condition may also be due to the high potassium content in the soil of the Tibetan plateau [49]. Excessive potassium fertilization can hinder plant growth [50] and lead to symptoms such as plant collapse [51,52]. The mechanism needs to be further investigated.

This result is mainly because for traditional Chinese medicine which uses roots or rhizomes as medicine, roots, as storage organs, are gradually consumed in the process of plant growth and development [53]. Therefore, these medicinal materials are mostly harvested in the dormant period. According to the results of this study, it is suggested to harvest *A. tanguticus* in the mature stage considering the yield needs of *A. tanguticus*.

### 3.2. Effects of Different Fertilization Methods and Harvest Periods on Alkaloid Content and Yield

The content of active ingredients is related to the quality of herbal medicines, and fertilization can effectively improve the content of secondary metabolic yield of herbal medicines and improve the quality of herbal medicines. There is a parabolic relationship between the fertilization rates of single nitrogen, phosphorus, and potassium fertilization of *A. tanguticus*, yield, and effective components. Within a certain fertilization range, yield and economic benefits both increase with the increase in fertilization rate but decrease beyond this range. This phenomenon is consistent with “the law of diminishing returns” [44]. The contents of anisodine, anisodamine, scopolamine, and atropine mostly showed a trend of increasing and then decreasing with increasing fertilization levels under different levels of N, P, and K treatments, and the highest content mostly occurred under the medium level of fertilization treatment, N2P2K2 treatment, which had the highest content of all four alkaloids of *A. tanguticus*.

From the greening stage to the growing stage, the growth rate of roots of *A. tanguticus* is faster and the yield increases rapidly, while from the growing stage to the maturity stage, the growth rate of roots is slower [41]. This is the same as the growth pattern of most tuberous medicinal plants such as Radix Codonopsis and Salvia, and the alkaloid yield also increased with the harvesting period, peaking at the maturity stage [16]. This is the same as the results of previous studies on the cultivation of *A. tanguticus*; the plant reaches its highest alkaloid yield in October. October is usually chosen as the time of collection for local use [32]. This is mainly because the harvesting of root and rhizome herbs takes place during the dormant period, as these storage organs are depleted during the growth and development of the plant [54].

### 3.3. Exploration of A. tanguticus Fertilizer Effect Model

For a long time, China’s “3414” test results are often fitted by the ternary quadratic fertilizer effect model. In recent years, most of the research on the fertilizer effect of “3414” has been carried out on crops, while there are relatively few studies on traditional Chinese medicine [37,42]. The results of this study showed that the goodness of fit of the ternary quadratic and binary quadratic fertilizer effect equations was between 0.68 and 0.92, and the equation was successfully fitted. The fitting analysis of the yield and alkaloid content of *A. tanguticus* at different harvesting periods showed that the yield of *A. tanguticus* was proportional to the increase in fertilizer application within a certain range, while the yield was significantly suppressed with the increase in fertilizer application after exceeding the maximum limit amount, indicating that the fitted equation was a typical fertilizer effect equation, which was consistent with the principle of diminishing returns. According to the analysis of the results, the fitted optimum yield and optimum quality values of NPK fertilization were higher than the two-factor fitted values, which indicated that the combined fertilization of NPK was more effective in the theoretical situation. Based on the fitted optimum fertilization rates, it was found that annual mature *A. tanguticus* was more suitable for growing under fertilization treatments with medium to high levels of nitrogen, medium to high levels of phosphorus, and medium to low levels of potassium. The second-year *A. tanguticus* was more suitable for fertilization with medium to high levels of nitrogen, medium to low levels of phosphorus, and low levels of potassium. Combined with the results of yield and alkaloid content, the fertilizer application rates of 225–300 kg/ha^2^ for N, 850–960 kg/ha^2^ for P, in 65–85 kg/ha^2^ for K are suggested.

## 4. Materials and Methods

### 4.1. Site Description

Huanzhong District, Qinghai Province, China, was selected as a sample site for the cultivation of *A. tanguticus* (36°47′7.08″ N, 101°30′49.30′′ E). The average altitude is 2480 m, and the terrain is high in the west and low in the east, with four distinct seasons, abundant water resources, annual average sunshine hours of 2588.3 h, annual average temperature of 0–5 °C, annual average precipitation of 360–650 mm, and annual evaporation of 900–1000 mm. We measured the indicators of the soil in the cultivated area without fertilization (Table 5).

### 4.2. Materials and Experimental Design

For the exploration of the *A. tanguticus* fertilizer effect model, growing seedlings were raised in Zhaojia Village, Nianbo Town, Ledu District, Haidong City, Qinghai Province. Seedlings with the same appearance and shape were transplanted in late April 2020, Weeds were manually cleared every second month. After the seedlings are harvested, diseased plants were identified and destroyed. Irrigation and pest management were the same as conventional farmland management. The trial was conducted in May 2020 in Huangzhong County, Qinghai Province, with healthy, pest-free, and mechanically undamaged *A. tanguticus* seedlings cultivated at a spacing of 40 cm × 50 cm and a plot area of 8.2 × 8.2 m. The fertilizer was urea (N) for N fertilizer, calcium superphosphate (P) for phosphorus fertilizer, and potassium sulfate (K) for potassium fertilizer. The experiment was a three-year continuous trial, and the plots were tested in “3414” randomized group experimental design; this is a field trial that should be performed in randomized complete block design (RCBD), with 3 factors (N, P, and K), 4 fertilization levels, and 14 treatments in total. To ensure the accuracy of the experiment, each treatment was replicated three times, and there were 42 plots in total. Each time the samples were collected, 5 plants were taken in each plot, and each treatment was equivalent to 15 replications, which ensured the accuracy of the experiment (Table 6). Fertilizers were applied in May every year.

### 4.3. Apparatus and Measurement Parameters

Four samples were collected each in October 2020, June 2021, August 2021, and October 2021. After collection, samples were cleaned, dried, and then weighed to obtain the dry weight. The material was then crushed with a crusher and passed through a 65 mesh sieve. The yield was determined separately for the aerial dry weight (electronic balance) and root dry weight (electronic balance). Alkaloid composition measurement involved the determination of anisodine, anisodamine, scopolamine, and atropine (HPLC).

Determination of alkaloid indexes: Two grams of anisodica medicinal powder was selected via a Mettler Toledo XS204 balance (Mettler Toledo Instruments Co., Ltd. Zurich, Switzerland). Then, 4 mL of ammonia water was added to it and mixed evenly. The mixture was allowed to stand for 10 min, after which 100 mL of chloroform was added, and the total weight was accurately weighed. It was subjected to ultrasound for 30 min, cooled to room temperature, and weighed; makeup for the loss of chloroform was added, and the mixture was filtered through cotton wool. Then 100 mL of the filtrate was taken and evaporated to dryness by rotary evaporation, the residue was dissolved in methanol and transferred to a 5 mL volumetric flask, and the mobile phase was filtered with a 0.45 μm filter membrane before use. An Agilent Technologies 1200 series high-performance liquid chromatograph and a Hypersil BDS C18 chromatographic column (Dalian Elite Analytical Instrument Co., Ltd., Dalian, China) were used. The reference substances for scopolamine hydrobromide, anisodine hydrobromide, anisodamine hydrobromide, and atropine sulfate were all from the China National Institute for the Control of Pharmaceutical and Biological Products. Methanol was chromatographically pure; triethylamine, tetrahydrofuran, glacial acetic acid, and sodium acetate were all analytical grade (Shanghai Chemical Reagent Factory, Shanghai, China); and the Milli-Q ultrapure water system was used for the pure water (Millipore, Burlington, MA, USA).

### 4.4. Data Processing and Analysis

#### 4.4.1. Alkaloid Content Calculation

The concentration of reference standards (C_r_) = sample weight (mg) × purity × coefficient ÷ volume (mL)

$$Content\%=\frac{C_{r}\times A_{s}\times 200\times 10}{m_{s}\times {\bar{A}}_{r}\times 1000\times 100\times (1-moisture\%)}\times 100\%$$

*Cr*—the concentration of the reference substance (mg/mL); *A_s_*—peak area of the sample; *m*—weight of sample (g); ${\bar{A}}_{r}$—average peak area of reference.

#### 4.4.2. Data Analysis

A factorial analysis of variance (ANOVA) was performed to evaluate the variance components and Duncan’s multiple range test was performed for mean multiple comparisons (α = 0.05) using SPSS 22.0 (Chicago, IL, USA) for the effect of N, P, and K fertilizer. The results were expressed as mean ± standard error (Mean ± SE), significance level α = 0.05. The effect of different nitrogen, phosphorus, and potassium fertilization conditions on important indicators of *A. tanguticus* was analyzed by range analysis. Graphs were constructed with Origin 2018 (Systat Software, Inc., Washington, DC, USA) software. Two- and three-factor equations were simulated using nonlinear regression to calculate optimal fertilization ratios and maximum yield/content.

## 5. Conclusions

The “3414” fertilization treatment was carried out on 1-year-old and 2-year-old *A. tanguticus*. The results showed that the application of N, P, and K significantly increased the alkaloid content and accumulation. Biomass accumulation and alkaloid content increased first and then decreased with the increase in N, P, and K. The maximum biomass accumulation of *A. tanguticus* was observed when N and P fertilizer application was at a medium level and K application was at medium and low levels (T6 and T9). The alkaloid content increased between October of the first year and June of the second year and decreased in the second year with the increase in the harvesting period. In contrast, *A. tanguticus* yield and alkaloid production decreased between October of the first year and June of the second year and increased with the increase in the harvesting period in the second year. The final proposed application rates of 225–300 kg/ha^2^ for nitrogen, 850–960 kg/ha^2^ for phosphorus, and 65–85 kg/ha^2^ for potassium were obtained by binary quadratic and ternary quadratic analysis. The optimal fertilization treatments obtained in this study will be further verified and validated in subsequent studies.

## Figures and Tables

**Table 1:** Effects of fertilizer interaction at different harvest periods on yield (kg) (In Table 1, 2020-10, 2021-6, 2021-8, 2021-10 represent October 2020, June 2021, August 2021 and October 2021 respectively. T1-T14 represents 14 fertilization methods. Different letters in columns indicate significant differences according to Duncan’s test at α = 0.05.).

Treatment	Sample Collection Time							
	2020-10		2021-6		2021-8		2021-10	
	Dry Weight Underground	Dry Weight above Ground	Dry Weight Underground	Dry Weight above Ground	Dry Weight Underground	Dry Weight above Ground	Dry Weight Underground	Dry Weight above Ground
T1	0.08 ± 0.035 d	0.13 ± 0.011 c	0.054 ± 0.007 c	0.058 ± 0.016 e	0.183 ± 0.097 c	0.171 ± 0.028 d	0.296 ± 0.084 e	0.146 ± 0.057 c
T2	0.1 ± 0.041 cd	0.15 ± 0.007 bc	0.064 ± 0.008 bc	0.078 ± 0.037 de	0.280 ± 0.090 abc	0.198 ± 0.029 bcd	0.458 ± 0.107 dce	0.187 ± 0.036 bc
T3	0.15 ± 0.105 ab	0.16 ± 0.011 bc	0.074 ± 0.012 bc	0.106 ± 0.031 bcd	0.311 ± 0.104 ab	0.269 ± 0.048 ab	0.529 ± 0.189 cd	0.200 ± 0.034 bc
T4	0.12 ± 0.043 bcd	0.16 ± 0.009 abc	0.068 ± 0.008 bc	0.087 ± 0.031 cde	0.280 ± 0.088 abc	0.197 ± 0.029 bcd	0.452 ± 0.219 dce	0.199 ± 0.026 bc
T5	0.12 ± 0.066 bcd	0.16 ± 0.01 bc	0.069 ± 0.010 bc	0.096 ± 0.036 bcd	0.302 ± 0.132 ab	0.209 ± 0.049 bcd	0.571 ± 0.209 bcd	0.218 ± 0.021 abc
T6	0.15 ± 0.065 a	0.2 ± 0.01 a	0.075 ± 0.008 bc	0.124 ± 0.043 ab	0.383 ± 0.094 a	0.321 ± 0.032 a	0.653 ± 0.137 ab	0.274 ± 0.021 ab
T7	0.11 ± 0.086 bcd	0.14 ± 0.013 bc	0.079 ± 0.008 bc	0.083 ± 0.032 cde	0.351 ± 0.210 a	0.266 ± 0.030 abc	0.527 ± 0.154 cd	0.169 ± 0.022 c
T8	0.1 ± 0.039 cd	0.15 ± 0.009 bc	0.087 ± 0.009 abc	0.107 ± 0.037 bcd	0.283 ± 0.106 abc	0.206 ± 0.027 bcd	0.581 ± 0.253 bcd	0.210 ± 0.016 abc
T9	0.12 ± 0.047 bcd	0.18 ± 0.009 ab	0.113 ± 0.023 a	0.113 ± 0.031 bc	0.289 ± 0.140 abc	0.261 ± 0.029 abc	0.691 ± 0.177 a	0.291 ± 0.025 a
T10	0.12 ± 0.038 bcd	0.16 ± 0.011 abc	0.069 ± 0.008 bc	0.095 ± 0.036 bcd	0.216 ± 0.070 bc	0.202 ± 0.043 bcd	0.419 ± 0.160 de	0.185 ± 0.026 c
T11	0.13 ± 0.076 bcd	0.18 ± 0.012 ab	0.095 ± 0.009 ab	0.146 ± 0.033 a	0.277 ± 0.080 abc	0.264 ± 0.042 abc	0.469 ± 0.215 dce	0.213 ± 0.016 abc
T12	0.13 ± 0.043 bcd	0.18 ± 0.014 ab	0.069 ± 0.008 bc	0.100 ± 0.047 bcd	0.287 ± 0.156 abc	0.212 ± 0.026	0.651 ± 0.352 bc	0.207 ± 0.022 abc
T13	0.13 ± 0.043 bcd	0.18 ± 0.01 ab	0.073 ± 0.012 bc	0.108 ± 0.054 bcd	0.216 ± 0.101 ab	0.179 ± 0.033 d	0.511 ± 0.299 cd	0.206 ± 0.020 abc
T14	0.12 ± 0.049 bcd	0.18 ± 0.013 ab	0.072 ± 0.008 bc	0.103 ± 0.027 bcd	0.204 ± 0.141 bc	0.190 ± 0.045 cd	0.493 ± 0.358 cd	0.207 ± 0.026 abc

**Table 2:** Effect of fertilizer interaction on alkaloid content at different harvest stages (%) (In Table 2, 2020-10, 2021-6, 2021-8, 2021-10 represent October 2020, June 2021, August 2021 and October 2021 respectively. T1-T14 represents 14 fertilization methods. Different letters in columns indicate significant differences according to Duncan’s test at α = 0.05).

Treatment	Sample Collection Time															
	2020-10				2021-6				2021-8				2021-10			
	Anisodine	Anisodamine	Scopolamine	Atropine	Anisodine	Anisodamine	Scopolamine	Atropine	Anisodine	Anisodamine	Scopolamine	Atropine	Anisodine	Anisodamine	Scopolamine	Atropine
T1	0.22 ± 0.011 g	0.05 ± 0.008 d	0.31 ± 0.012 ef	0.34 ± 0.018 e	0.272 ± 0.031 d	0.118 ± 0.009 bc	0.245 ± 0.021 d	0.553 ± 0.031 d	0.163 ± 0.041 e	0.025 ± 0.005 e	0.272 ± 0.028 e	0.366 ± 0.027 f	0.213 ± 0.021 de	0.037 ± 0.004 e	0.214 ± 0.037 e	0.223 ± 0.016 e
T2	0.29 ± 0.008 fg	0.05 ± 0.01 cd	0.42 ± 0.019 bc	0.4 ± 0.012 de	0.328 ± 0.023 cd	0.124 ± 0.008 abc	0.218 ± 0.035 cd	0.622 ± 0.020 cd	0.203 ± 0.021 e	0.046 ± 0.010 cde	0.275 ± 0.028 e	0.525 ± 0.073 bcdef	0.258 ± 0.016 cde	0.038 ± 0.007 de	0.338 ± 0.015 cde	0.276 ± 0.015 de
T3	0.34 ± 0.016 abcd	0.1 ± 0.015 abc	0.58 ± 0.026 b	0.59 ± 0.019 ab	0.450 ± 0.013 abc	0.144 ± 0.007 abc	0.319 ± 0.020 bcd	0.842 ± 0.047 ab	0.312 ± 0.061 bcde	0.122 ± 0.003 ab	0.362 ± 0.030 cde	0.623 ± 0.035 abcd	0.283 ± 0.023 bcde	0.063 ± 0.003 bcd	0.394 ± 0.011 bcd	0.371 ± 0.023 bcd
T4	0.29 ± 0.017 cdef	0.06 ± 0.013 cd	0.48 ± 0.017 de	0.36 ± 0.022 e	0.343 ± 0.033 bcd	0.124 ± 0.004 abc	0.321 ± 0.026 bcd	0.672 ± 0.057 bcd	0.222 ± 0.053 de	0.050 ± 0.010 cde	0.356 ± 0.025 cde	0.453 ± 0.042 def	0.277 ± 0.009 bcde	0.053 ± 0.004 cde	0.297 ± 0.025 cde	0.300 ± 0.027 cde
T5	0.33 ± 0.023 abcde	0.1 ± 0.015 ab	0.57 ± 0.025 bc	0.39 ± 0.022 de	0.378 ± 0.034 abcd	0.136 ± 0.001 abc	0.338 ± 0.024 bcd	0.802 ± 0.068 abc	0.252 ± 0.039 de	0.078 ± 0.010 abc	0.455 ± 0.026 abcde	0.644 ± 0.057 abcd	0.308 ± 0.026 bcd	0.060 ± 0.005 bcde	0.452 ± 0.029 abc	0.304 ± 0.040 cde
T6	0.41 ± 0.012 a	0.14 ± 0.008 a	0.68 ± 0.019 a	0.62 ± 0.027 a	0.532 ± 0.020 a	0.193 ± 0.002 a	0.540 ± 0.026 a	0.878 ± 0.038 ab	0.483 ± 0.028 a	0.127 ± 0.003 a	0.639 ± 0.021 a	0.754 ± 0.067 a	0.398 ± 0.020 a	0.081 ± 0.007 a	0.536 ± 0.046 a	0.503 ± 0.027 a
T7	0.35 ± 0.014 abc	0.09 ± 0.018 bcd	0.62 ± 0.023 ab	0.59 ± 0.02 ab	0.365 ± 0.022 bcd	0.148 ± 0.002 abc	0.347 ± 0.024 bcd	0.597 ± 0.032 cd	0.359 ± 0.052 abcd	0.073 ± 0.001 cde	0.524 ± 0.028 abcd	0.605 ± 0.097 abcde	0.233 ± 0.028 de	0.057 ± 0.002 bcde	0.306 ± 0.022 cde	0.325 ± 0.031 bcde
T8	0.27 ± 0.009 efg	0.08 ± 0.011 bcd	0.58 ± 0.029 b	0.48 ± 0.04 bcd	0.368 ± 0.021 bcd	0.133 ± 0.012 abc	0.398 ± 0.024 ab	0.737 ± 0.027 bcd	0.406 ± 0.043 abc	0.083 ± 0.007 abc	0.427 ± 0.025 abcde	0.563 ± 0.100 abcdef	0.280 ± 0.015 bcde	0.065 ± 0.001 bc	0.503 ± 0.023 ab	0.437 ± 0.056 abc
T9	0.38 ± 0.004 ab	0.11 ± 0.02 ab	0.67 ± 0.018 a	0.58 ± 0.011 abc	0.503 ± 0.039 ab	0.173 ± 0.003 ab	0.412 ± 0.029 ab	1.008 ± 0.109 a	0.427 ± 0.024 ab	0.090 ± 0.006 abc	0.603 ± 0.022 ab	0.694 ± 0.077 abc	0.370 ± 0.024 abc	0.074 ± 0.008 bc	0.516 ± 0.023 a	0.463 ± 0.051 ab
T10	0.28 ± 0.004 def	0.05 ± 0.009 bcd	0.57 ± 0.017 bc	0.45 ± 0.021 de	0.350 ± 0.019 bcd	0.097 ± 0.007 c	0.270 ± 0.047 bcd	0.744 ± 0.061 bcd	0.284 ± 0.038 bcde	0.082 ± 0.006 abc	0.364 ± 0.020 cde	0.532 ± 0.048 bcdef	0.175 ± 0.022 e	0.053 ± 0.005 cde	0.336 ± 0.023 cde	0.277 ± 0.035 de
T11	0.3 ± 0.013 bcdef	0.07 ± 0.006 bcd	0.59 ± 0.017 b	0.46 ± 0.026 cde	0.353 ± 0.022 bcd	0.129 ± 0.001 abc	0.280 ± 0.009 bcd	0.756 ± 0.033 bcd	0.228 ± 0.022 de	0.076 ± 0.002 bcd	0.588 ± 0.025 abc	0.710 ± 0.074 ab	0.312 ± 0.028 bcd	0.065 ± 0.001 bc	0.386 ± 0.028 bcd	0.385 ± 0.060 bcd
T12	0.31 ± 0.026 bcdef	0.11 ± 0.012 ab	0.56 ± 0.026 b	0.6 ± 0.053 ab	0.456 ± 0.042 abc	0.121 ± 0.001 bc	0.395 ± 0.021 ab	0.875 ± 0.059 ab	0.306 ± 0.036 bcde	0.061 ± 0.007 cde	0.426 ± 0.022 abcde	0.501 ± 0.037 cdef	0.288 ± 0.034 bcde	0.058 ± 0.001 bcde	0.376 ± 0.027 cde	0.350 ± 0.041 bcde
T13	0.36 ± 0.007 abc	0.12 ± 0.03 a	0.6 ± 0.026 b	0.49 ± 0.045 bcd	0.367 ± 0.027 bcd	0.130 ± 0.002 abc	0.385 ± 0.022 abc	0.778 ± 0.146 bc	0.268 ± 0.044 cde	0.055 ± 0.006 cde	0.384 ± 0.029 bcde	0.412 ± 0.032 ef	0.358 ± 0.026 abc	0.079 ± 0.001 b	0.230 ± 0.024 de	0.380 ± 0.062 bcd
T14	0.35 ± 0.009 abcd	0.05 ± 0.005 cd	0.51 ± 0.008 cd	0.46 ± 0.014 cde	0.333 ± 0.023 cd	0.119 ± 0.020 bc	0.180 ± 0.024 bcd	0.706 ± 0.063 bcd	0.195 ± 0.023 e	0.030 ± 0.005 de	0.313 ± 0.047 de	0.412 ± 0.053 ef	0.383 ± 0.019 ab	0.068 ± 0.007 bc	0.327 ± 0.022 cde	0.355 ± 0.048 bcde

**Table 3:** Range analysis of effect of N-P-K fertilization on main indexes (In Table 3, 2020-10, 2021-6, 2021-8, 2021-10 represent October 2020, June 2021, August 2021 and October 2021 respectively).

Main Index		Range Value			Fertilizer Effect Ordination
		N	P	K	
2020-10	Anisodine	0.1832	0.2075	0.3232	K > P > N
	Anisodamine	0.2532	0. 2436	0.2488	N > K > P
	Scopolamine	0.0460	0.0532	0.0261	P > N > K
	Atropine	0.1023	0.1956	0.1875	P > K > N
	Aboveground production	0.2923	0.6981	0.7065	K > P > N
	Underground production	0.2452	0.4541	0.5621	K > P > N
2021-6	Anisodine	0.1220	0.1100	0.1247	K > N > P
	Anisodamine	0.0301	0.0310	0.0710	N > P > K
	Scopolamine	0.1558 i	0.0920	0.1642	P > K > N
	Atropine	0.1821	0.2540	0.1057	P > K > N
	Aboveground production	0.0300	0.0152	0.0262	N > K > P
	Underground production	0.0644	0.0300	0.0191	N > P > K
2021-8	Anisodine	0.1796	0.1060	0.2540	K > N > P
	Anisodamine	0.0560	0.0497	0.1060	N > P > K
	Scopolamine	0.1280	0.2788	0.2375	P > K > N
	Atropine	0.1371	0.2090	0.1885	P > K > N
	Aboveground production	0.0640	0.0510	0.0590	N > K > P
	Underground production	1.1880	0.5730	0.4960	N > P > K
2021-10	Anisodine	0.0868	0.1347	0.2246	K > P > N
	Anisodamine	0.0323	0.0246	0.0308	N > K > P
	Scopolamine	0.1630	0.2410	0.2246	P > N > K
	Atropine	0.0490	0.2962	0.1740	P > K > N
	Aboveground production	0.3943	0.6718	0.7189	K > P > N
	Underground production	0.4553	0.6310	0.7474	K > P > N

**Table 4:** NPK fertilizer response equation and recommended fertilizer rates for optimal *A. tanguticus* growth.

	Model	Nutrient	Fertilizer Response Equation	Maximum Rate (g·Plant^−1^)	Maximum Production	R^2^
Anisodine (%)	Binary	N	y = −0.043 + 0.002 * N + 0.001 * P − (2.402 × 10^−6^) * N^2^ − (−2.772 × 10^−7^) * P^2^ − (8.537 × 10^−7^) * N * P	265.153	0.378	0.92
		P		597.835		
		N	y = 0.1 + 0.001 * N + 0.03 * K − (2.782 × 10^−6^) * N^2^ − (1.406 × 10^−5^) * K^2^ − (1.005 × 10^−6^) * N * K	226.388	0.398	0.83
		K		75.984		
		P	y = 0.078 + 0.0004 * N + 0.03 * K − (3.1 × 10^−7^) * N^2^ − (1.428 × 10^−5^) * K^2^ − (4.013 × 10^−7^) * N * K	602.71	0.405	0.72
		K		80.12		
	Ternary	N	y = 0.21 + 0.001 * N + 0.0002 * P + 0.001 * K − (1.809 × 10^−6^) * N^2^ − (2.4 × 10^−7^) * P^2^ − (1.175 × 10^−5^) * k^2^ − (3.299 × 10^−7^) * N * P + (3.16 × 10^−6^) * N * K + (7.881 × 10^−7^) * P * K	222.807	0.405	0.91
		P		683.182		
		K		84.900		
Anisodamine (%)	Binary	N	y = −0.045 + 0.001 * N + 0.0001 * P − (1.099 × 10^−6^) * N^2^ − (6.83 × 10^−8^) * P^2^ − (2.108 × 10^−7^) * N * P	288.727	0.077	0.85
		P		948.276		
		N	y = 0.03 + 0.001 * N + 0.004 * K − (1.144 × 10^−6^) * N^2^ − (2.535 × 10^−6^) * K^2^ + (6.088 × 10^−7^) * N * K	280.385	0.079	0.73
		K		159.343		
		P	y = −0.03 + 0.0001 * N + 0.001 * K − (−6.81 × 10^−8^) * N^2^ − (2.422 × 10^−6^) * K^2^ − (4.048 × 10^−7^) * N * K	874.133	0.089	0.89
		K		109.598		
	Ternary	N	y = 0.36 + 0.0001 * N + (9.834 × 10^−5^) * P + (5.212 × 10^−5^) * K − (8.355 × 10^−6^) * N^2^ − (5.162 × 10^−7^) * P^2^ − (1.835 × 10^−5^) * k^2^ − (1.02 × 10^−7^) * N * P + (1.908 × 10^−6^) * N * K − (1.01 × 10^−7^) * P * K	225.421	0.081	0.91
		P		890.069		
		K		75.507		
Scopolamine (%)	Binary	N	y = −0.148 + 0.003 * N + 0.001 * P − (4.151 × 10^−6^) * N^2^ − (4.329 × 10^−7^) * P^2^ − (1.353 × 10^−6^) * N * P	235.692	0.500	0.81
		P		679.795		
		N	y = −0.867 + 0.007 * N + 0.009 * K − (4.852 × 10^−6^) * N^2^ − (9.828 × 10^−6^) * K^2^ − (3.511 × 10^−5^) * N * K	226.387	0.511	0.79
		K		143.000		
		P	y = 0.078 + 0.0004 * N + 0.03 * K − (3.1 × 10^−7^) * N^2^ − (1.428 × 10^−5^) * K^2^ − (4.013 × 10^−7^) * N * K	715.716	0.419	0.82
		K		103.127		
	Ternary	N	y = 0.189 + 0.0003 * N + 0.002 * P + 0.003 * K − (1.021 × 10^−6^) * N^2^ − (2.387 × 10^−7^) * P^2^ − (1.478 × 10^−5^) * k^2^ + (1.887 × 10^−7^) * N * P − (8.205 × 10^−6^) * N * K − (1.133 × 10^−6^) * P * K	295.638	0.534	0.86
		P		854.064		
		K		67.135		
Atropine (%)	Binary	N	y = 0.482−0.001 * N + 0.0001 * P + (1.06 × 10^−6^) * N^2^ − (1.06 × 10^−7^) * P^2^ + (1.438^−7^) * N * P	171.690	0.342	0.78
		P		486.268		
		N	y = 0.116 + 0.004 * N + 0.02 * K − (1.4 × 10^−6^) * N^2^ − (4.043 × 10^−6^) * K^2^ − (1.748 × 10^−6^) * N * K	226.387	0.310	0.81
		K		99.984		
		P	y = 0.117 + 0.0004 * N + 0.02 * K − (1.362 × 10^−7^) * N^2^ − (4.015 × 10^−6^) * K^2^ − (1.771 × 10^−6^) * N * K	960.000	0.403	0.79
		K		120.981		
	Ternary	N	y = 0.28 + 0.001 * N + 0.0002 * P + 0.02 * K − (6.642 × 10^−6^) * N^2^ − (1.896 × 10^−7^) * P^2^ − (5.634 × 10^−5^) * k^2^ + (1.344 × 10^−7^) * N * P + (2.404 × 10^−6^) * N * K − (1.773 × 10^−7^) * P * K	300.214	0.505	0.85
		P		930.203		
		K		66.782		
Production (kg)	Binary	N	y = 0.555 − 0.001 * N + 0.0001 * P + (6.122 × 10^−6^) * N^2^ − (3.037 × 10^−7^) * P^2^ + (1.544 × 10^−6^) * N * P	145.445	0.657	0.68
		P		585.092		
		N	y = −0.494−0.007 * N + 0.09 * K − (8.09 × 10^−6^) * N^2^ − (1.927 × 10^−5^) * K^2^ − (2.459 × 10^−5^) * N * K	162.1548	0.685	0.82
		K		102.890		
		P	y = 0.61 + 0.002 * N + 0.1 * K − (4.78 × 10^−7^) * N^2^ − (2.103 × 10^−5^) * K^2^ − (6.88 × 10^−6^) * N * K	608.9	0.689	0.72
		K		118.32		
	Ternary	N	y = 0.277 + 0.00001 * N + (3.456 × 10^−5^) * P + 0.006 * K − (5.21 × 10^−6^) * N^2^ − (2.464 × 10^−7^) * P^2^ − (1.257 × 10^−5^) * k^2^ + (3.548 × 10^−6^) * N * P + (8.519 × 10^−6^) * N * K − (2.643 × 10^−7^) * P * K	235.84	0.695	0.75
		P		964.28		
		K		85.19		

**Table 5:** Soil indicators in cultivated areas without fertilization.

Indicator	Value	Unit
Total nitrogen	2.10	g/kg
Total phosphorus	0.88	g/kg
Total potassium	17.23	g/kg
Organic matter	18.85	g/kg
PH	7.27	
Electrical conductivity	226.67	

**Table 6:** Fourteen different experimental fertilizer treatments based on the “3414” optimal design scheme.

No.	Number	Fertilization Treatment	Fertilizer Rates (kg/ha^2^)		
			N	P	K
T1	1, 28, 38	N0P0K0	0	0	0
T2	2, 27, 39	N0P2K2	0	900	150
T3	3, 26, 35	N1P2K2	112.5	900	150
T4	4, 23, 40	N2P0K2	225	0	150
T5	5, 25, 37	N2P1K2	225	450	150
T6	6, 24, 36	N2P2K2	225	900	150
T7	7, 22, 31	N2P3K2	225	1350	150
T8	8, 19, 32	N2P2K0	225	900	0
T9	9, 21, 34	N2P2K1	225	900	75
T10	10, 18, 42	N2P2K3	225	900	225
T11	11, 20, 29	N3P2K2	337.5	900	150
T12	12, 17, 41	N1P1K2	112.5	450	150
T13	13, 16, 33	N1P2K1	112.5	900	75
T14	14, 15, 30	N2P1K1	225	450	75

## Data Availability

The data presented in this study supporting the results are available in the main text. Additional data are available upon reasonable request from the corresponding author.

## References

[1] Xie Z.W. (1975). National Chinese Herbal Medicine Compilation.

[2] Zhao H.Y., Zhou Q.M., Zhu H., Zhou F., Meng C.W., Shu H.Z., Liu Z.H., Peng C., Xiong L. (2021). Anisotanols A–D, Four Norsesquiterpenoids with an Unprecedented Sesquiterpenoid Skeleton from *Anisodus tanguticus*. Chin. J. Chem..

[3] Wei Z., Wang L., Meng L., Liu J. (2008). Genetic variation in the endangered *Anisodus tanguticus* (Solanaceae), an alpine perennial endemic to the Qinghai-Tibetan Plateau. Genetica.

[4] Wu Z.Y., Raven P.H., Hong D.Y. (1994). Flora of China.

[5] Yang Y.C. (1991). Flora Tebitan Medicine.

[6] Zhang G., Chi X. (2019). The complete chloroplast genome of *Anisodus tanguticus*, a threatened plant endemic to the Qinghai-Tibetan Plateau. Mitochondrial DNA Part B.

[7] Chen C., Wang B., Li J., Xiong F., Zhou G. (2022). Multivariate Statistical Analysis of Metabolites in *Anisodus tanguticus (Maxim.) Pascher* to Determine Geographical Origins and Network Pharmacology. Front. Plant Sci..

[8] Jiang Y.B., Zhong M., Hu M.X., Chen L., Gou Y., Zhou J., Wu P.E., Ma Y.Y. (2017). Spectrum-effect relationships between high-performance liquid chromatography fingerprint and analgesic property of *Anisodus tanguticus* (Maxim) Pascher (Solanaceae) roots. Trop. J. Pharm. Res..

[9] Yang D.Z., Zhang Z.Y., Lu A.M., Sun K., Liuc J.Q. (2002). Floral organogenesis and development of two taxa of the Solanaceae—*Anisodus tanguticus* and *Atropa belladonna*. Isr. J. Plant Sci..

[10] He T., Jia J.F. (2009). Breaking dormancy in seeds of *Anisodus tanguticus*: An endangered medicinal herb of high altitude in the Qinghai-Tibet Plateau. Seed Sci. Technol..

[11] Tao L., Ping Z., Ke C., Chao M., Hui H. (2005). Molecular cloning, expression and characterization of hyoscyamine 6beta-hydroxylase from hairy roots of *Anisodus tanguticus*. Planta Med..

[12] Guo H., Wu X., Wang A., Luo X., Ma Y., Zhou M. (2015). Separation and detection of tropane alkaloids in *Anisodus tanguticus* by capillary electrophoresis-electrochemiluminescence. New J. Chem..

[13] Li X., Li W., Zhao M., Zhang T. (2021). Application of anisodamine in pediatrics. Heilongjiang Med. Sci..

[14] Lei T., Cai X., Wang H., Li S., Shen J., Zhou D. (2016). Progress in biosynthesis mechanism and bioengineering of tolane alkaloids. J. Northwest. Bot..

[15] She S., Ma J., Zhang F., Liu P. (2022). Effect of gastrodin combined with anisodine hydrobromide on neurological function in elderly patients with cognitive dysfunction of small cerebral vascular disease. Eval. Anal. Hosp. Drugs China.

[16] Jingyu Z., Yajing L., Zhongyi Z., Chaofei Y., Xiaotong G. (2018). Molecular Regulation of Catalpol and Acteoside Accumulation in Radial Striation and non-Radial Striation of Rehmannia glutinosa Tuberous Root. Int. J. Mol. Sci..

[17] He Y., Cui G., Feng Z., Jie Z., Li Y. (2004). Conservation priorities for plant species of forest-meadow ecotone in Sanjiangyuan Nature Reserve. Chin. J. Appl. Ecol..

[18] Grunert O., Hernandez-Sanabria E., Buysens S., Neve S.D., Boon N. (2020). In-Depth Observation on the Microbial and Fungal Community Structure of Four Contrasting Tomato Cultivation Systems in Soil Based and Soilless Culture Systems. Front. Plant Sci..

[19] Geng G., Wang G., Stevanato P., Lv C., Wang Y. (2021). Physiological and Proteomic Analysis of Different Molecular Mechanisms of Sugar Beet Response to Acidic and Alkaline pH Environment. Front. Plant Sci..

[20] Du X.Q., Wang F.L., Li H., Jing S., Yu M., Li J., Wu W.H., Kudla J., Wang Y. (2019). The Transcription Factor MYB59 Regulates K+/NO3-Translocation in the Arabidopsis Response to Low K+ Stress. Plant Cell.

[21] Berthod N., Brereton N.J., Pitre F.E., Labrecque M. (2015). Five willow varieties cultivated across diverse field environments reveal stem density variation associated with high tension wood abundance. Front. Plant Sci..

[22] Yang Z.-J., Wu X.-H., Grossnickle S.C., Chen L.-H., Yu X.-X., El-Kassaby Y.A., Feng J.-L. (2020). Formula Fertilization Promotes Phoebe bournei Robust Seedling Cultivation. Forests.

[23] Kazimierczak R., Średnicka-Tober D., Barański M., Hallmann E., Góralska-Walczak R., Kopczyńska K., Rembiałkowska E., Górski J., Leifert C., Rempelos L. (2021). The Effect of Different Fertilization Regimes on Yield, Selected Nutrients, and Bioactive Compounds Profiles of Onion. Agronomy.

[24] Rubio L., García-Pérez D., García-Sánchez M.J., Fernández J.A. (2018). Na^+^-Dependent High-Affinity Nitrate, Phosphate and Amino Acids Transport in Leaf Cells of the Seagrass *Posidonia oceanica* (L.) Delile. Mol. Sci..

[25] Ju X.T., Kou C.L., Christie P., Dou Z.X., Zhang F.S. (2007). Changes in the soil environment from excessive application of fertilizers and manures to two contrasting intensive cropping systems on the North China Plain. Environ. Pollut..

[26] Yang Z.J., Wu X.H., Chen L.H., Huang L.M., Chen Y., Wu J., El-Kassaby Y.A., Grossnickle S.C., Feng J.L. (2021). Fertilization Regulates Accumulation and Allocation of Biomass and Nutrients in Phoebe bournei Seedlings. Agriculture.

[27] Chen Y., Zhou X., Lin Y., Ma L. (2019). Pumpkin Yield Affected by Soil Nutrients and the Interactions of Nitrogen, Phosphorus, and Potassium Fertilizers. HortScience.

[28] Pasqualone A., Summo C., De Angelis D., Cucci G., Caranfa D., Lacolla G. (2021). Effect of Mineral and Organic Fertilization on desi and kabuli Chickpea (*Cicer arietinum* L.): Plant Growth and Production, Hydration Properties, Bioactive Compounds, and Antioxidant Activity. Plants.

[29] Duan Y., Zhang T., Liu J. (2007). Pollination biology of Anisodus tanguticus (Solanaceae). Biodivers. Sci..

[30] Zeng H., Wang X., Qiao Z., Li Y., Cai N., Liu S., Li Y. (2017). Influence on Yield and Quality of Lonicera japonica by Soil Testing and Formulated Fertilization. J. Nucl. Agric. Sci..

[31] Han X.J., Zhang X.Z. (2014). Status and Changing Trend of Soil Nutrient Contents in Cultivated Land from the Implementation of Soil Test and Formula Fertilization in Wuhu County Anhui Province. Chin. J. Soilence.

[32] Zhang M., Li J., Kong Q., Yan F. (2016). Progress and prospect of the study on crop-response-to-fertilization function model. Acta Pedol. Sin..

[33] Szczepanek M., Siwik-Ziomek A. (2019). P and K Accumulation by Rapeseed as Affected by Biostimulant under Different NPK and S Fertilization Doses. Agronomy.

[34] Chen Z., Ma H., Xia J., Hou F., Shi X., Hao X., Hafeez A., Han H., Luo H. (2017). Optimal pre-plant irrigation and fertilization can improve biomass accumulation by maintaining the root and leaf productive capacity of cotton crop. Sci. Rep..

[35] Chen B., Wang Q., Ye Z., Stiles S., Feng G. (2020). Optimisation of phosphorus fertilisation promotes biomass and phosphorus nutrient accumulation, partitioning and translocation in three cotton (*Gossypium hirsutum*) genotypes. Crop Pasture Sci..

[36] Jeda P., Thorburn P.J., Biggs J.S., Dominati E.J., Probert M.E., Meier E.A., Huth N.I., Mike D., Val S., Larsen J.R. (2017). Nitrogen Cycling from Increased Soil Organic Carbon Contributes Both Positively and Negatively to Ecosystem Services in Wheat Agro-Ecosystems. Front. Plant Sci..

[37] Wang K., Zhang R., Song L., Yan T. (2020). Comparison of C:N:P stoichiometry in the plant–litter–soil system between poplar and elm plantations in the Horqin Sandy Land, China. Front. Plant Sci..

[38] Gu X., Ding M., Lu W., Lu D. (2019). Nitrogen topdressing at the jointing stage affects the nutrient accumulation and translocation in rainfed waxy maize. J. Plant Nutr..

[39] Liu Y., Ma W., He H., Wang Z., Cao Y. (2021). Effects of Sugarcane and Soybean Intercropping on the Nitrogen-Fixing Bacterial Community in the Rhizosphere. Front. Microbiol..

[40] Guo S., Xiong W., Hang X., Gao Z., Jiao Z., Liu H., Mo Y., Zhang N., Kowalchuk G.A., Li R. (2021). Protists as main indicators and determinants of plant performance. Microbiome.

[41] Du J., Shen T., Xiong Q., Zhu C., Chen X. (2020). Combined proteomics, metabolomics and physiological analyses of rice growth and grain yield with heavy nitrogen application before and after drought. BMC Plant Biol..

[42] Ghaley B.B., Wösten H., Olesen J.E., Schelde K., Baby S., Karki Y.K., Børgesen C.D., Smith P., Yeluripati J., Ferrise R. (2018). Simulation of Soil Organic Carbon Effects on Long-Term Winter Wheat (*Triticum aestivum*) Production under Varying Fertilizer Inputs. Front. Plant Sci..

[43] Ren B., Guo Y., Liu P., Zhao B., Zhang J. (2021). Effects of Urea-Ammonium Nitrate Solution on Yield, N_2_O Emission, and Nitrogen Efficiency of Summer Maize Under Integration of Water and Fertilizer. Front. Plant Sci..

[44] Ballard T., Yeo G., Neal A., Farrell S. (2016). Departures from Optimality When Pursuing Multiple Approach or Avoidance Goals. J. Appl. Psychol..

[45] Chapman H.D. (1973). Plant analysis values suggestive of nutrient status of selected crops. Soil Testing and Plant Analysis. Part 2. Plant Analysis.

[46] Grossnickle S.C. (2000). Ecophysiology of Northern Spruce Species The Performance of Planted Seedlings. Tree Physiology..

[47] Lv Z., Lu G. (2021). A New Curve of Critical Leaf Potassium Concentration Based on the Maximum Root Dry Matter for Diagnosing Potassium Nutritional Status of Sweet Potato. Front. Plant Sci..

[48] Cba B., Jy A., Bo C.C., Ying X.A., Pg A., Hui L.A., Gl A. (2020). Growth years and post-harvest processing methods have critical roles on the contents of medicinal active ingredients of Scutellaria baicalensis—ScienceDirect. Ind. Crop Prod..

[49] Wan D.S., Feng J.J., Jiang D.C., Mao K.S., Duan Y.W., Miehe G., Opgenoorth L. (2016). The Quaternary evolutionary history, potential distribution dynamics, and conservation implications for a Qinghai–Tibet Plateau endemic herbaceous perennial, *Anisodus tanguticus* (Solanaceae). Ecol. Evol..

[50] Liu L., Zuo Z.T., Xu F.R., Wang Y.Z. (2020). Study on Quality Response to Environmental Factors and Geographical Traceability of Wild Gentiana rigescens Franch. Front. Plant Sci..

[51] Sharma A., Sharma B., Hayes S., Kerner K., Hoecker U., Jenkins G.I., Franklin K.A. (2019). UVR8 disrupts stabilisation of PIF5 by COP1 to inhibit plant stem elongation in sunlight. Nat. Commun..

[52] Luo C., Guo Z., Xiao J., Dong K., Dong Y. (2021). Effects of Applied Ratio of Nitrogen on the Light Environment in the Canopy and Growth, Development and Yield of Wheat When Intercropped. Front. Plant Sci..

[53] Melo E., Gonçalves J., Rocha J., Hakamada R., Bazani J., Wenzel A., Arthur J., Borges J., Malheiros R., Lemos C. (2016). Responses of Clonal Eucalypt Plantations to N, P and K Fertilizer Application in Different Edaphoclimatic Conditions. Forests.

[54] Mi S., Zhang X., Wang Y., Ma Y., Sang Y., Wang X. (2022). Effect of different fertilizers on the physicochemical properties, chemical element and volatile composition of cucumbers. Food Chem..

